# AI‐based staging, causal hypothesis and progression of subjects at risk of Alzheimer's disease: a multicenter study

**DOI:** 10.1002/alz70856_105530

**Published:** 2026-01-09

**Authors:** Simona Aresta, Raffaello Nemni, Moreno Zanardo, Graziella Sirabian, Dario Capelli, Marco Alì, Paolo Vitali, Enrico Giuseppe Bertoldo, Valentina Fiolo, Lilla Bonanno, Giuseppa Maresca, Petronilla Battista, Francesco Sardanelli, Francesca B Pizzini, Isabella Castiglioni, Christian Salvatore, The Alzhemer's Disease Neuroimaging Initiative (ADNI)

**Affiliations:** ^1^ University School for Advanced Studies IUSS Pavia, Italy, Pavia, Italy; ^2^ Istituti Clinici Scientifici Maugeri IRCCS, Bari, ‐, Italy; ^3^ Bracco Imaging ‐ Centro Diagnostico Italiano – Milano, Milan, Italy; ^4^ Department of Biomedical Sciences for Health ‐ Università degli Studi di Milano, Milan, Italy; ^5^ Clinical Psychology Service ‐ Centro Diagnostico Italiano, Milan, Italy; ^6^ Centro Diagnostico Italiano ‐ Bracco Imaging, Milan, Italy; ^7^ Unit of Radiology, IRCCS Policlinico San Donato & Department of Biomedical Sciences for Health ‐ Universita' degli Studi di Milano, Milan, Italy; ^8^ Clinical Psychology Service ‐ IRCCS Policlinico San Donato Milan San Donato Milanese, Milan, Italy; ^9^ IRCCS Centro Neurolesi Bonino Pulejo, Messina, Italy; ^10^ Maugeri Clinical Inistitute IRCCS, Bari, Italy; ^11^ Università degli Studi di Milano, Milano, Italy; ^12^ IRCCS Policlinico San Donato, San Donato Milanese, Italy; ^13^ Neuroradiology, Department of Diagnostics and Pathology, Verona University Hospital, Verona, Italy; ^14^ DeepTrace Technologies S.r.l., Milan, Italy; ^15^ Università Milano Bicocca, Milan, Italy; ^16^ IUSS, Pavia, Italy; ^17^ ADNI, Washington D.C., DC, USA

## Abstract

**Background:**

In 2024, eleven European scientific societies/organizations and one patient advocacy association have defined a patient‐centered biomarker‐based diagnostic workflow for memory clinics evaluating neurocognitive disorders. This study aimed to evaluate the clinical performance of an Artificial Intelligence (AI)‐tool applied to neuropsychological assessment and MRI for supporting the staging, clinical profiling, diagnosis, causal hypothesis, and progression of subjects at risk of Alzheimer's disease (AD) following the above‐mentioned intersocietal recommendations.

**Method:**

This observational, multicentric study enrolled 796 subjects: 705 from the Alzheimer's Disease Neuroimaging Initiative (ADNI) database, 35 from Centro Diagnostico Italiano (Italy), 26 from IRCCS Policlinico San Donato (Italy), and 30 from IRCCS Bonino Pulejo (Italy). Participants were clinically staged as healthy subjects (HS), subjective cognitive impairment (SCI), mild cognitive impairment (MCI), or AD‐dementia at baseline and 24‐month follow‐up.

Patients were clinically profiled into AD clinical syndromes based on cognitive characteristics and structural neuroimaging findings. First‐line biomarkers were also measured. The AI‐based software TRACE4AD™ automatically processed neuroimaging and neuropsychological test data to extract cognitive and structural findings. The tool staged subjects as HS/SCI, MCI, or moderate‐to‐severe‐dementia (MSD), profiled the causal hypothesis, and predicted conversion risk to AD‐dementia.

Agreement between AI and human staging was assessed using Cohen's kappa. AI‐performance was evaluated by sensitivity, specificity, positive predictive value (PPV), negative predictive value (NPV), area under curve (AUC), and accuracy.

**Result:**

For the staging classification the inter‐rater AI‐humans agreement was substantial for both HS/SCI vs. rest (Cohen's κ = 0.81) and MCI (κ = 0.70) classification, almost perfect for MSD vs. rest (κ = 0.90) classification. For the causal hypothesis classification, the AI performance vs. biomarker‐based diagnosis was: PPV 91%, NPV 100%, and accuracy 91%. For the binary classification of progression to AD‐dementia at 24‐month, the AI performance was: sensitivity 89%, specificity 82%, accuracy 85%, and AUC 83%.

**Conclusion:**

The AI‐tool demonstrated its usefulness in supporting the clinical treatment of AD patients by assisting with staging, clinical profiling, diagnosis, hypothesis generation for underlying causes, and predicting the risk of progression to AD‐related dementia within 24 months.

